# The Effect of Farmers’ Decisions on Pest Control with *Bt* Crops: A Billion Dollar Game of Strategy

**DOI:** 10.1371/journal.pcbi.1004483

**Published:** 2015-12-31

**Authors:** Alice E. Milne, James R. Bell, William D. Hutchison, Frank van den Bosch, Paul D. Mitchell, David Crowder, Stephen Parnell, Andrew P. Whitmore

**Affiliations:** 1 Computational and Systems Biology, Rothamsted Research, Harpenden, United Kingdom; 2 Rothamsted Insect Survey, Rothamsted Research, Harpenden, United Kingdom; 3 Department of Entomology, University of Minnesota, St. Paul, Minnesota, United States of America; 4 Department of Agricultural and Applied Economics, University of Wisconsin, Madison, Wisconsin, United States of America; 5 Department of Entomology, Washington State University, Pullman, Washington, United States of America; 6 School of Environment & Life Sciences, University of Salford, Manchester, United Kingdom; 7 Sustainable Soils and Grassland Systems, Rothamsted Research, Harpenden, United Kingdom; University of New South Wales, AUSTRALIA

## Abstract

A farmer’s decision on whether to control a pest is usually based on the perceived threat of the pest locally and the guidance of commercial advisors. Therefore, farmers in a region are often influenced by similar circumstances, and this can create a coordinated response for pest control that is effective at a landscape scale. This coordinated response is not intentional, but is an emergent property of the system. We propose a framework for understanding the intrinsic feedback mechanisms between the actions of humans and the dynamics of pest populations and demonstrate this framework using the European corn borer, a serious pest in maize crops. We link a model of the European corn borer and a parasite in a landscape with a model that simulates the decisions of individual farmers on what type of maize to grow. Farmers chose whether to grow *Bt*-maize, which is toxic to the corn borer, or conventional maize for which the seed is cheaper. The problem is akin to the snow-drift problem in game theory; that is to say, if enough farmers choose to grow *Bt* maize then because the pest is suppressed an individual may benefit from growing conventional maize. We show that the communication network between farmers’ and their perceptions of profit and loss affects landscape scale patterns in pest dynamics. We found that although adoption of *Bt* maize often brings increased financial returns, these rewards oscillate in response to the prevalence of pests.

## Author Summary

A farmer’s decision on whether to control a pest is usually based on the perceived threat of the pest locally and the guidance of commercial advisors. Therefore, farmers in a region are often influenced by similar circumstances, and this can create a coordinated response to a pest. This coordinated response, although not intentional, can affect ecological systems at the landscape scale. Using the European corn borer as an exemplar system, we develop a framework to explore the feedback mechanisms between pest populations and farmers’ decisions. We show that the form of communication network and the farmers’ perceptions of profit and loss influence the decisions made on pest control. Our work has implications for other systems, whereby the ecology of a system is driven by individual decision makers following similar heuristics and experiencing similar influences. Indeed, by understanding the feedback mechanisms between pest populations and farmers’ decisions we can predict landscape-scale dynamics and determine how to manipulate these to sustain control.

## Introduction

**Fig 1:**
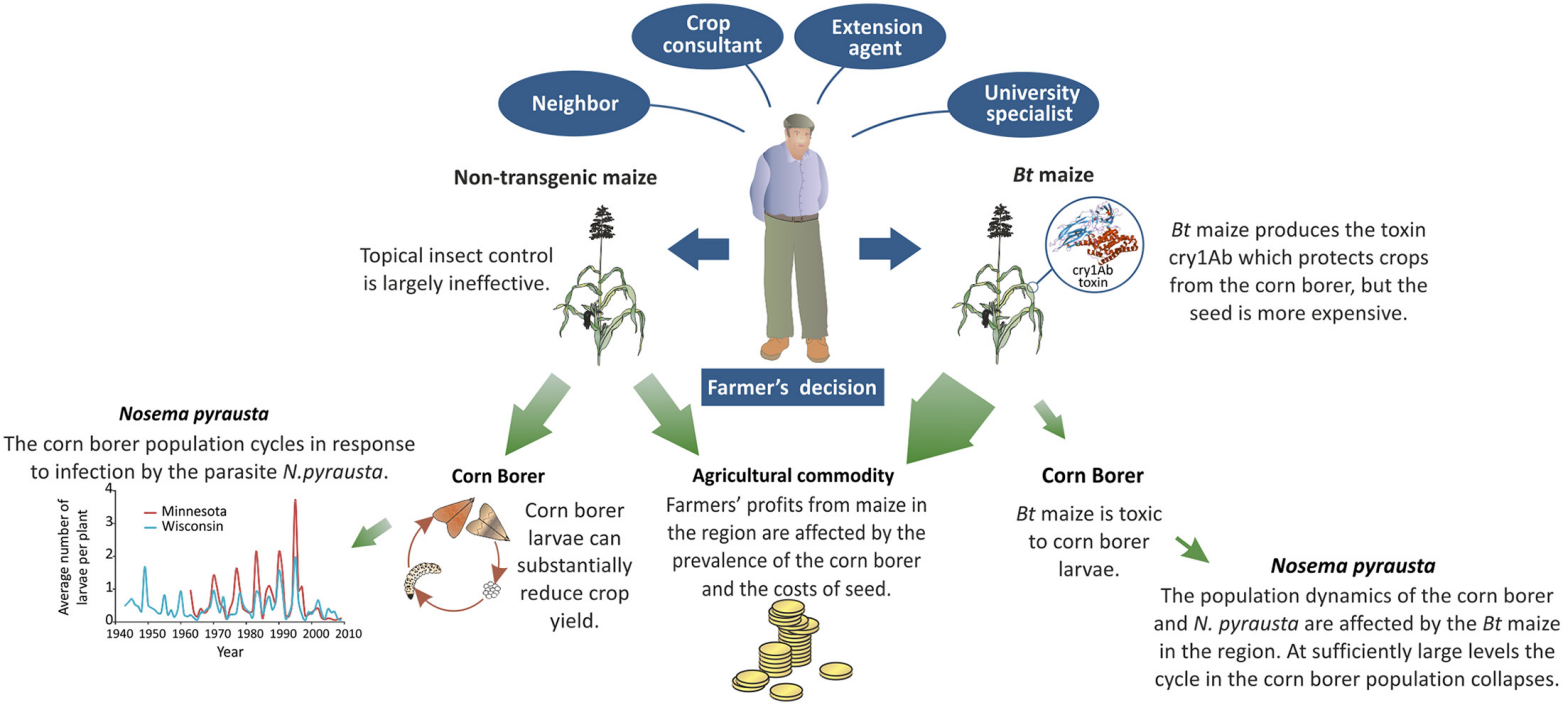
Influences on farmers’ decisions and their impacts. A schematic illustrating the influences on farmers' decisions on what varieties of maize to grow, and how this impacts the population dynamics of the European corn borer and the profitability of farming at a landscape scale. The width of the green arrows indicates the approximate appropriation of agricultural resources.

The European corn borer (*Ostrinia nubilalis*) (ECB), a serious pest of maize, cost the American economy an estimated 1 billion US dollars annually at its worst in the early 1990s [1, 2]. In 1996, *Bt* maize, a transgenic crop that expressed insecticidal proteins from the soil-dwelling bacterium *Bacillus thuringiensis*, was introduced for control of the pest. Since then, farmers have had to choose whether to plant conventional or *Bt* maize (Fig 1). Their decisions rest on the economic viability of *Bt*, given that future infestations of ECB cannot be predicted. Specifically, farmers must predict whether increased returns from *Bt* will exceed the technology fee, a financial premium for buying the transgenic seed [3, 4]. In some situations, farmers believe that the economics favor conventional seed; more than half of them believe that the price of *Bt* maize is too high to merit purchase [1, 5], particularly if their crops have not recently been infested.

Hutchison *et al*. [1] showed that *Bt* maize generated an estimated $230 million annual benefit to maize growers in Illinois, Minnesota and Wisconsin. Much of this economic benefit (75%) accrued to farmers who did not plant *Bt* maize; these farmers did not pay technology fees but still benefitted from the area-wide suppression provided by those farmers who cooperated to use *Bt* to reduce pest densities [1]. Other systems, such as cotton, have shown similar benefits from area wide suppression of pests [6].

As such, the control of ECB can be evaluated through game theory because the mechanisms of cooperation, such as reciprocity, reputation and spatial structure are embedded in the farmer networks that mediate the population dynamics of the pest [7–10]. The system is akin to a ‘snow drift’ game [8]. The snow drift game is a metaphor for a situation whereby the benefit that an individual, in this case a farmer, obtains for a given strategy depends on the actions of others. In particular, if a farmer chooses to grow conventional maize in a landscape where the pest is supressed by other farmers growing *Bt* maize, then this individual will benefit from the pest suppression without paying the technology fee. On the other hand, in a situation where the pest is not suppressed at landscape scale it is likely to be more profitable for an individual to grow *Bt* maize.

When deciding whether to plant *Bt* maize, farmers negotiate between ‘expert’ and ‘local’ knowledge (Fig 1). For example, Kaup’s [5] hierarchy of influences showed maize-seed dealers and crop consultants appeared to have substantial influence, neighbors had moderate influence, and extension agents had little influence on the farmers’ decisions to plant *Bt* maize. More than 50% of farmers who anticipated having ECB problems chose to plant *Bt* maize. The results emphasize an important principle in pest control: farmers’ perceived risks, rather than actual losses, play an important role in pest management [5, 11, 12]. This principle of 'risk perception' is crucial. If farmers’ underestimate the risk of infestation and grow conventional maize then the pest will flourish and diminish yields. If on the other hand farmers exaggerate the risk and plant too much *Bt* maize then there is an increased risk that the pest will adapt to its new host and threaten the long-term production of maize.

Here we build a framework for exploring the intrinsic feedback mechanisms between the actions of humans and the dynamics of pest populations in a structured landscape, and use the European corn borer in maize as an example. Our example is intended to demonstrate the plausibility of the framework and so is illustrative rather than predictive. Our models are kept simple to both aid the elucidation of our results and to reduce the runtimes of the simulations. This particular example was chosen because there is a rich source of data to support it. We build a mechanistic model of the population dynamics of ECB in a 700-km long strip of the US Corn Belt. The models are parameterised to reflect a maize system similar to that in the part of the US Corn Belt that passes through Minnesota and Wisconsin. The model of the population dynamics includes the life cycle, dispersal and ecology of the pest including its relationship with the pathogen *Nosema pyrausta* (Microsporidia: Nosematidae), which is one of the most important natural enemies of the ECB; this parasite reduces the number of surviving offspring, and is cited as the primary reason for the observed cycle in the population density [13–16]. The landscape model is spatially-explicit and parameterized so that one half has similar county sizes, farm sizes, and density of maize crops to those in Minnesota and the other to those in Wisconsin. We show how this model captures the behavior of the ECB-population dynamics in the observed empirical data at a coarse spatial scale. Importantly, analysis of the model shows that even when the infected population is reduced to small numbers, it retains the capacity to recover and so the natural control persists.

We then introduce a sociological layer to the model. We simulate the processes by which individual farmers decide whether to grow *Bt* maize or conventional maize. The decision is based predominantly on likely profit: the probability that a farmer will chose a given strategy is based on the information that he or she has on the profits achieved under *Bt* maize and conventional maize in recent seasons. For any given farmer, the source of this information will depend on the network of communication. Here we explicitly model four different networks of communication. In particular we explore how the form of the network affects the uptake of *Bt* maize over time, the pest population dynamics and the long term profits of the farmers in the landscape. We show that the form of the network impacts the feedback mechanism between pest populations and farmers' decisions that affect landscape-scale dynamics. We show that independent decision makers that follow similar heuristics and are influenced by the same circumstances can create an apparent coordinated response which affects ecological systems at landscape scales. This coordinated response is not intentional, but is an emergent property of the system.

## Methods

Below we present the components of the model framework, including the pest dynamics model, the farmer decision model and four different communication networks. We then use this framework to explore the effect of the different communication networks and the responsiveness of the farmers to loss on (i) the pest dynamics, (ii) the uptake of *Bt* maize and (iii) the long term losses of the farmers.

### European corn borer and *Nosema pyrausta* model

We developed a model to explore the population dynamics of ECB and its natural enemy, the pathogen *Nosema pyrausta*, and the impact of ECB on maize crops in a landscape. This landscape was based on national agricultural census statistics from 1997, 2002 and 2007 on county sizes, farm sizes and numbers, harvested areas and the area of maize grown in Wisconsin and Minnesota [17–19]. We used a grid of 300 x 1400 cells that equates to a 150km x 700km strip. Each cell represents 25 ha (0.5km x 0.5km), similar to the typical size of maize fields in the region. One half of the simulated landscape was parameterised to be similar to Wisconsin and the other to Minnesota. We partitioned the two states into counties, with county sizes reflecting the actual distribution of county sizes in each state. We defined farms as connected cells in which arable crops could be grown. The number of farms in each simulated county, and the distribution of their sizes, reflected the true distribution of arable land on farms in each state. Simulated farms were fitted into the county, along with uncropped areas at random (see S1 Text). The landscape was generated stochastically and so is a realisation of a random process.

Crops were assigned county by county. On average, maize accounted for 44% of the cropped area in Minnesota and 37% in Wisconsin [17–19]. Cropped cells were then allocated at random as maize or other. Each year, the proportion of maize in a given county was resampled, and cropped cells allocated again at random to maize or other. This process allowed for a proportion of fields to have maize crops grown consecutively and others to have rotations with a non-host crop for ECB. We made the simplifying assumption that ECB only develops in grid cells with maize. In each of these cells we use an abundance-based population model to describe the development of a population of ECB that is susceptible to the pathogen *N*. *pyrausta* and one that is infected. Our model did not include the effect of other natural enemies of ECB or climate, and so was not expected to accurately describe the historic dynamics of the ECB. Rather, its purpose was to capture the population cycle attributed to *N*. *pyrausta* and to simulate the effect of *Bt* maize on larval survival.

In the model, eggs hatch into larvae that pass through five instar stages. The survival of the larvae through to pupation is density dependent. We assume that the *Bt* toxin reduces the number of larvae that reach instar 3 by 99.9% [20]. We do not consider insecticides as a control measure as these are considered largely ineffective because after the neonate stage, the ECB larvae are concealed within the maize plant, thus avoiding direct contact with an insecticide's active ingredients. Adults emerge following pupation, then disperse and mate, and then females disperse before oviposition and the cycle starts again. We assume two generations of ECB per year, as is typical in Minnesota and Wisconsin. The larvae from the second generation overwinter in stalks, and so their survival rate is lower than that of the first generation. Infection by *N*. *pyrausta* travels through both horizontal and vertical pathways. We assume that infected adult males do not pass infection to their young, but that females pass on infection to 85% of their eggs [21]. Infection passes horizontally through the population during the larvae stage when susceptible (uninfected) larvae come into contact with frass from infected larvae. The infection rate is modelled as density dependent. The survival of the infected population at each stage is smaller than the healthy population. The parameter values of the model were based on the body of work by Onstad and colleagues [12, 21, 22] (see S2 Text for full model description).

We modelled the dispersal of the populations in four stages: pre-mating dispersal, mating, post-mating dispersal of females, and oviposition. The dispersal functions represent the integration of the movement of moths over a period of days. The dispersal of insects is often modelled with an exponential dispersal kernel which has a mode at the origin. The literature [23–24] suggest that in the case of the corn borer, however, this may not be appropriate as instinct and environmental factors force large numbers of adults from their natal fields. For this reason, and for computational efficiency we chose to model dispersal using a beta distribution, which has a flexible mode. We assume dispersal is the same in all directions, and that at the boundary of the landscape the moths are reflected back.

**Fig 2:**
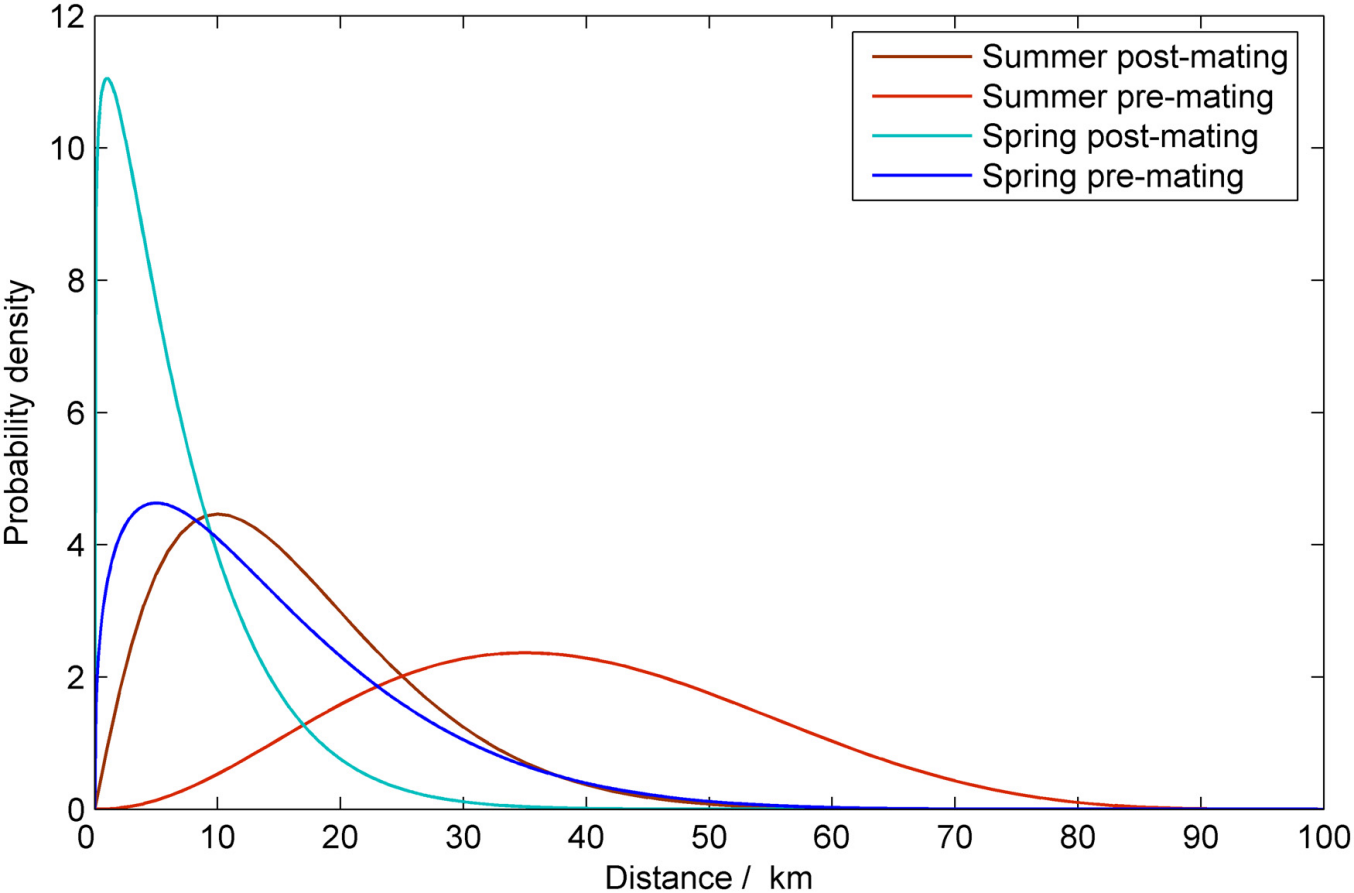
The functions used to model the dispersal of the European corn borer. The dispersal functions for adult moths pre- and post- mating in spring and summer.

We base our dispersal estimates on observations in the literature which demonstrate seasonal differences in the dispersal of spring and summer adults [23–26]. Crop rotation and lack of adequate humidity in crops during the day time can force newly emerged adults to move from their overwintering field before initiating sexual activity [27]. The probability density function (PDF) that describes the pre-mating dispersal in spring has a mode of 10km and 90% of the population travelling less than 30 km. The dispersal of infected moths is reduced by 80%. Dispersal in summer is more conservative with a mode of 1km and 90% of the adult moths fly less than 15km. Under typical conditions, the pre-oviposition period has a mean of 3.6 days [14]. Thereafter the mean oviposition period is approximately 10 days with oviposition decreasing with time. During this time a female could cover a considerable area. We assumed that for spring the mode of the post-mating PDF was 35 km and that 90% of the population travel less than 60 km, and that in summer the mode was 5 km with 90% of the population traveling less than 30 km (see Fig 2).

**Fig 3:**
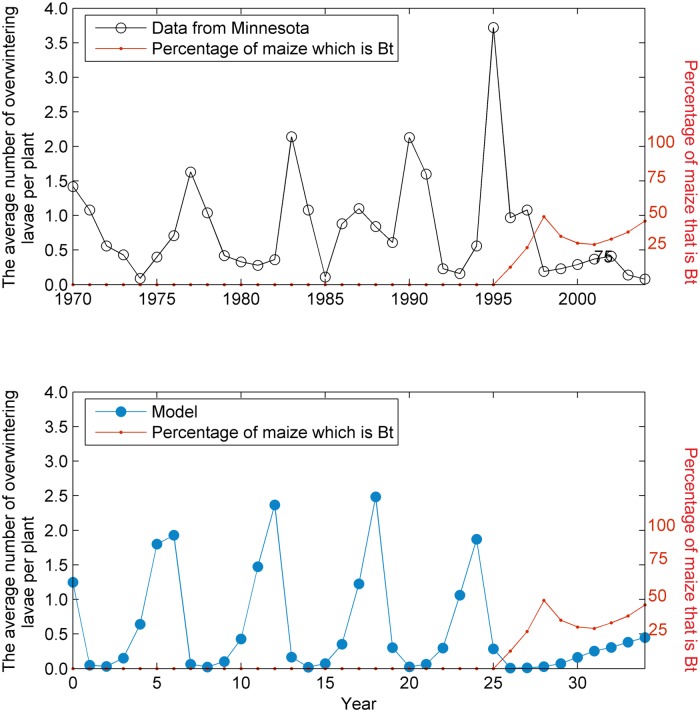
Overwintering larvae. Average numbers of overwintering lavae from Minnesota over time (solid black line) during a period where the proportion of *Bt* maize broadly increased (dashed red line). Our simulation model (solid blue line) captures the behavior observed in the field with a cycle in the population of similar wavelength to that observed in the data. The introduction of *Bt* maize results in this cycle being damped but still persisting (the cycle is under-damped in this case—see S2 Text).

The model of the ECB population density expresses the cycle of infestation caused by *N*. *pyrausta* observed in the field data with a similar wavelength [2]. When *Bt* was introduced into the landscape, the cycle collapsed and the pest was suppressed in a way similar to observed patterns [2] (Fig 3).

### Modelling the decision process

In the model, farmers growing maize face the decision of whether to plant *Bt* or conventional maize. As described above, the decisions on which type of maize to grow directly impacts the survival of the ECB larvae and so the population dynamics of the pest. Kaup [5] surveyed 4000 farmers in Wisconsin and Minnesota and found that the most common reasons for growing *Bt* maize were: (i) to increase yield; (ii) to control insects better; and (iii) they anticipated ECB problem. The most common reasons for not using *Bt* maize were (i) the price of *Bt* seed was too high; or (ii) no ECB problem was anticipated. Although growers may misconceive the financial impact of the drivers described above, these drivers imply a profit-based decision. Other factors including farm size, age, education and available market information have been shown to influence the adoption of GM crops and complex empirical models have been proposed to describe these effects on farmer decisions [28]. To both ensure the easy interpretation of our results, we chose to use a simple model based on perceived profit.

We assumed that the decision process is driven by the financial impact of ECB, and that farmers make decisions based on recent years’ experience [5]. We used data from Wisconsin and Minnesota on the estimated benefit ($ ha^−1^) from *Bt* maize and the increase in the area of *Bt* maize grown (as a percentage of total maize grown) between 1995 and 2009 to model the probability (*p*) of farmers changing cropping strategy (Hutchison et al., [1]). The following exponential function was used based on empirical and theoretical considerations:

$$\begin{array}{ll} p=1-\text{exp}\left[-\beta (r_{A}-r_{F})\right] & \text{where }r_{A}>r_{F}\\ p=0 & \text{otherwise} \end{array}.$$ (1)

Here *β* is a parameter, *r*_*F*_ is the reward the farmer perceives was attained under the chosen strategy and *r*_*A*_ is the reward the farmers perceives would have been attained under the alternative strategy, so that the difference *r*_*A*_−*r*_*F*_ measures the perceived net benefit for *Bt* maize adoption. This model is not only more parsimonious than a more traditional logistic model, but also has better goodness of fit criteria (see S1 Fig). Furthermore, the exponential model is a constant absolute risk aversion utility function for the representative farmer with parameters estimated to fit the observed state-level *Bt* maize adoption data and estimated benefit [29, 30]. The parameter *β* quantifies farmer responsiveness to the perceived gain from *Bt* maize adoption (or equivalently, ECB loss). The regression estimate for *β* was 0.0055 with a standard error of 0.00174 with no evidence to support separate parameters for each state. In practice it would be possible to influence farmer responsiveness (i.e. *β*) through subsidy, taxation or education. For example if farmers were encouraged to be cautious about returning to conventional maize then farmers growing *Bt* maize would be less responsive when they experienced an apparent benefit reduction. We used the fitted value ± three standard errors to define the range of values for *β* that we explored in our analysis.

For each season, we sample an individual farmer’s decision from a distribution whereby the probability of changing strategy is *p* (as defined in Eq 1). This allows us to implicitly include a range of individual behaviors from the intransigent farmer who finds a preferred strategy and will not change, to the receptive farmer who will try new practices. It also implicitly includes other social factors which we do not explicitly account for.

The farmer’s reward is given by the average financial reward from his maize fields calculated as

$$r=(Y-Y_{L})m_{P}-F,$$ (2)

where *Y* is the expected yield in a ECB-free crop (t ha^−1^), *Y*_*L*_ is the loss in yield due to the ECB (t ha^−1^), *m*_*p*_ is the crop price ($ t^−1^) and *F* is the technology fee ($), which is the seed price difference between conventional and *Bt* maize. We do not include varietal effects that could modify yields slightly, but assume that all maize crops have the same expected yield (10 t ha^−1^). We assume that this yield is reduced by ECB according to the function given in the supplementary information of Hutchison et al., [1]:

$$Y_{L}=Y\frac{0.021{\left(2.56x+5.65\sqrt{x}\right)}^{1.16}}{{\left[{\left(2.56x+5.65\sqrt{x}\right)}^{2}+{\left(3.4+1.73x\right)}^{2}\right]}^{0.29}},$$ (3)

where *x* is the average number of overwintering larvae per plant. To be consistent with the data used to parameterise the landscape model we assume *F* = 16 $ ha^−1^ and a crop price (*m*_*p*_) of 99 $ t^−1^ which are averages for Minnesota and Wisconsin between 1996 and 2009 [1].

### Communication networks

Given that we can calculate the reward (*r*) for growing maize in any particular field we must consider how to calculate the reward the farmer perceives was attained under each strategy (i.e.*r*_*F*_ and *r*_*A*_). The reward for a given strategy may be calculated from the rewards obtained for this strategy over a given area of the landscape, i.e. a farmer’s perceived reward depends on the network of communication and how much credence the farmer gives to the information available to them. Kaup [5] showed that growers who had reported an insect problem in one year were likely to grow *Bt* maize in the next, which is consistent with farmers who grow other *Bt* crops [31]. In Kaup’s study the state-reported insect levels did not significantly influence behavior. Therefore we assume that a farmer perceives that the reward for their chosen strategy (*r*_*F*_) is given by the average reward from across their fields, taking no account of the success of that strategy in their neighborhood.

To inform on the perceived reward from the alternative strategy we consider four networks of communication that we shall refer to as: (i) landscape-network; (ii) neighbor-network; (iii) Kaup-network and (iv) varying-response-network. There are two theoretical extremes: the first is where each farmer has information from across the whole landscape, akin to accessing web-based crop data. In this scenario the perceived reward for the alternative strategy is the average of the rewards for the alternative strategy across the landscape. We call this the ‘landscape-network’. The second is where each farmer has information only from farms that neighbor their own, which may reflect how traditional farming decisions are made alone or within cooperatives. In this scenario the reward for the alternative strategy is given by the average reward that this strategy attains in farms that neighbor the farmer. We call this the ‘neighbor-network’.

Research shows that when farmers decide which varieties to grow they may consult family and friends, other farmers, commercial newsletters, county extension agents and university specialists. Kaup [5] reports that 40.2% of farmers acknowledged that a major reason to grow *Bt* was that it was recommended by their seed dealers or consultants. Similarly 7.9% of farmers acknowledged recommendation by a neighbor, and 3.4% acknowledged recommendation by university or extension agencies. Normalizing these percentages to sum to 100%, we simulate a communication network whereby a farmer has a probability of 0.78 of being influenced by a consultant, a probability 0.15 of being influenced by a neighbour and a probability of 0.07 of being influenced by a university. According to those probabilities each farm is assigned a communication network type. For those assigned to be neighbor-influenced we calculate the reward of the alternative strategy by averaging the scores of this strategy from farms within 1km. We assume consultants operate over a county, and so for farmers assigned to be consultant-influenced we calculated the reward as the average reward across a county. Finally we assume universities operate at the state level and so the reward for those assigned to be university-influenced is given by the average reward across the state. This network, which we refer to as the ‘Kaup-network’, is arguably more common in today's farming environment than the two former scenarios. For each network we set the responsiveness parameter *β* (Eq 1) to 0.0055, 0.0003 and 0.0108, which are the value fitted to the data, and that value ± three standard errors.

Kaup [5] showed that if farmers had planted *Bt* in the past then they were more likely to use it in the future. This tendency is incorporated into the model by scaling β in Eq (1) so that farmers who have used *Bt* maize in the past are more responsive to loss of profit. Our final network, the ‘varying-response-network’, incorporates a reluctance for farmers to change back from *Bt*-maize to conventional maize. It assumes a Kaup-network with the probability of a farmer switching to *Bt* maize, having previously tried it given by Eq (1) with *β* = 0.0055 otherwise *β* = 0.0003.

### Implementing the model

We ran each simulation for 100 seasons. At the end of each season the reward *r*_F_(*i*) is calculated for each farm *i* along with the perceived reward for the alternative strategy *r*_A_(*i*). The probability that the farm strategy will change is calculated according to the farmer’s responsiveness to loss. This probability is used to determine if they change strategy. Crops are rotated and fields growing maize are assigned to *Bt* or conventional maize according to the calculated strategy.

## Results

### Analysis of the European corn borer and *Nosema pyrausta* model

To explore the behavior of the solutions of the model we considered the equations without the spatial component. Ignoring dispersal, the model equations (listed in S2 Text) reduce to the following set of difference equations:

$$\begin{array}{c} \tilde{S}(t)=\frac{a(S(t)+cP(t)){\text{e}}^{-\alpha P(t)}}{\nu +S(t)+P(t)}\\ \tilde{P}(t)=\frac{k[P(t)+b(S(t)+cP(t))(1-{\text{e}}^{-\alpha P(t)})]}{\nu +S(t)+P(t)}\\ S(t+1)=\omega_{1}\frac{a(\tilde{S}(t)+c\tilde{P}(t)){\text{e}}^{-\alpha \tilde{P}(t)}}{\nu +\tilde{S}(t)+\tilde{P}(t)}\\ P(t+1)=\omega_{2}\frac{k[\tilde{P}(t)+b(\tilde{S}(t)+c\tilde{P}(t))(1-{\text{e}}^{-\alpha \tilde{P}(t)})]}{\nu +\tilde{S}(t)+\tilde{P}(t)} \end{array}$$ (4)

where *S*(*t*) and *P*(*t*) represent the number of susceptible and infected eggs in year t, for the first generation respectively and $\tilde{S}(t)$ and $\tilde{P}(t)$ are for the second generation. The first pair of equations describes the summer generation and the second pair the autumn-spring generation. Many of the parameters result from combinations of biologically meaningful parameters from the full model (see S2). Parameters *a* = 929.8 and *k* = 85.6 capture the population increase from births modulated by survival rates for susceptible and healthy populations respectively. Parameter *c* = 0.15 is the proportion of susceptible eggs produced by an infected female. The term (1−e^−αP(t)^) determines the proportion of the healthy population that becomes infected, where α = 0.72 controls the infection transfer from the infected to susceptible population. Parameter *b* = 2.31 relates to the survival of this recently infected population. The carrying capacity parameter ν = 130.7 controls the density dependent survival of the larvae, parameters ω_1_ = 0.081 and ω_2_ = 0.02835 relate to the overwintering survival of the susceptible and infected populations respectively.

Analysis of these equations shows three steady-states, i.e. solutions where the rates of change of healthy population (*S*) and the infected population (*P*) are zero: (C1) [P* = 0, S* = 0], (C2) [P* = 0, $S*\;=\;\frac{a^{2}\omega_{1}-\nu^{2}}{a+\nu }$], and (C3) [P* = *P*_0_, S* = *S*_0_], where both *P*_0_ and *S*_0_ are positive real values. Linearization around these points determines the behavior of the solutions of the equations [32]. The first steady-state (C1) relates to the trivial solution whereby both healthy and infected populations become extinct; the second (C2) relates to the solution where the infected population becomes extinct; and the third steady-state (C3) relates to the solutions where both the healthy and the infected population densities are larger than zero and the total population cycles. It can be shown that (C3) exists, implying that *N*. *pyrausta* survives in the system, for parameter combinations such that $\sqrt{\omega_{2}}\left(\frac{k+\alpha b\hat{S}}{\nu +\hat{S}}\right)>1$, where $\hat{S}=\frac{a^{2}\omega_{1}-\nu^{2}}{a+\nu }$. For the model parameters used, and a wide range around these parameters, the steady-state (C3) always exists supporting the hypothesis that even if ECB is suppressed to low levels, the infected population will survive and the natural control given by *N*. *pyrausta* persists.

### The snow-drift game

**Fig 4:**
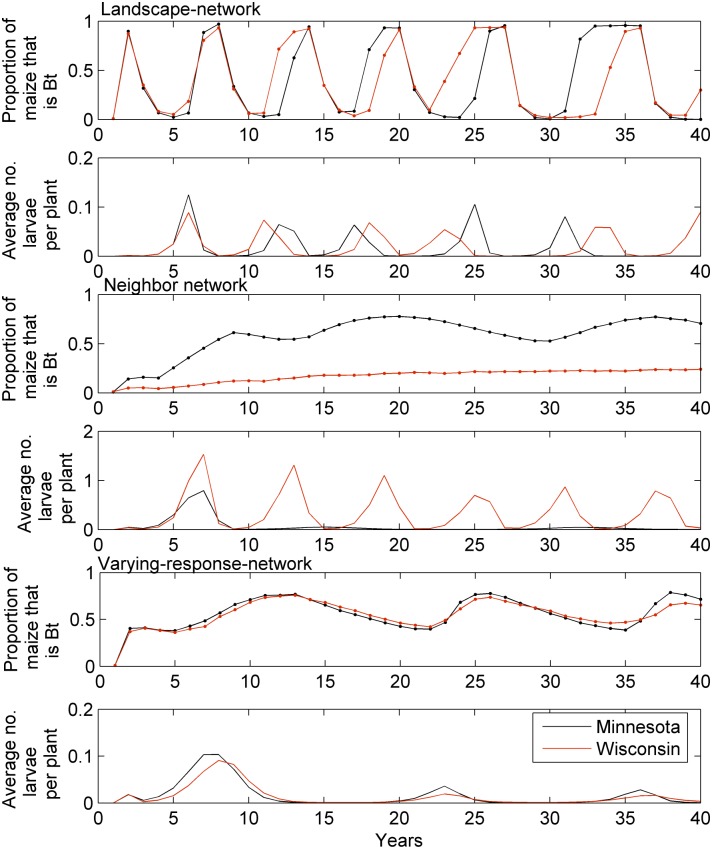
Results from the landscape-network, neighbor-network, and varying-response-network simulations. The top pane of each pair shows the proportion of *Bt* maize and bottom panes show the average number of overwintering larvae per plant across the two areas of the landscape, one in Wisconsin and the other in Minnesota. The simulation was started with 1% of the maize as *Bt* distributed randomly in the landscape.

Under the landscape-network simulation shown in Fig 4a and 4b, the percentage of *Bt* maize oscillates between approximately 1% and 95% over time. Larval populations are driven by the *Bt* adoption and oscillate similarly, with the largest levels prior to the maxima in the *Bt* cycle. Increasing farmer responsiveness to economic loss (i.e. increasing the parameter *β* in Eq 1) increases the frequency and amplitude of the oscillation; reducing farmer responsiveness reduces the frequency and amplitude of the oscillation. The average larval density is held near or below the economic threshold (0.06 larvae per plant for the model parameterization reported here), however, in some parts of the landscape the density was much higher. The results from the Kaup-network are similar to the landscape-network, but with a slightly higher oscillation frequency and slight dampening (see S2 Fig).

**Fig 5:**
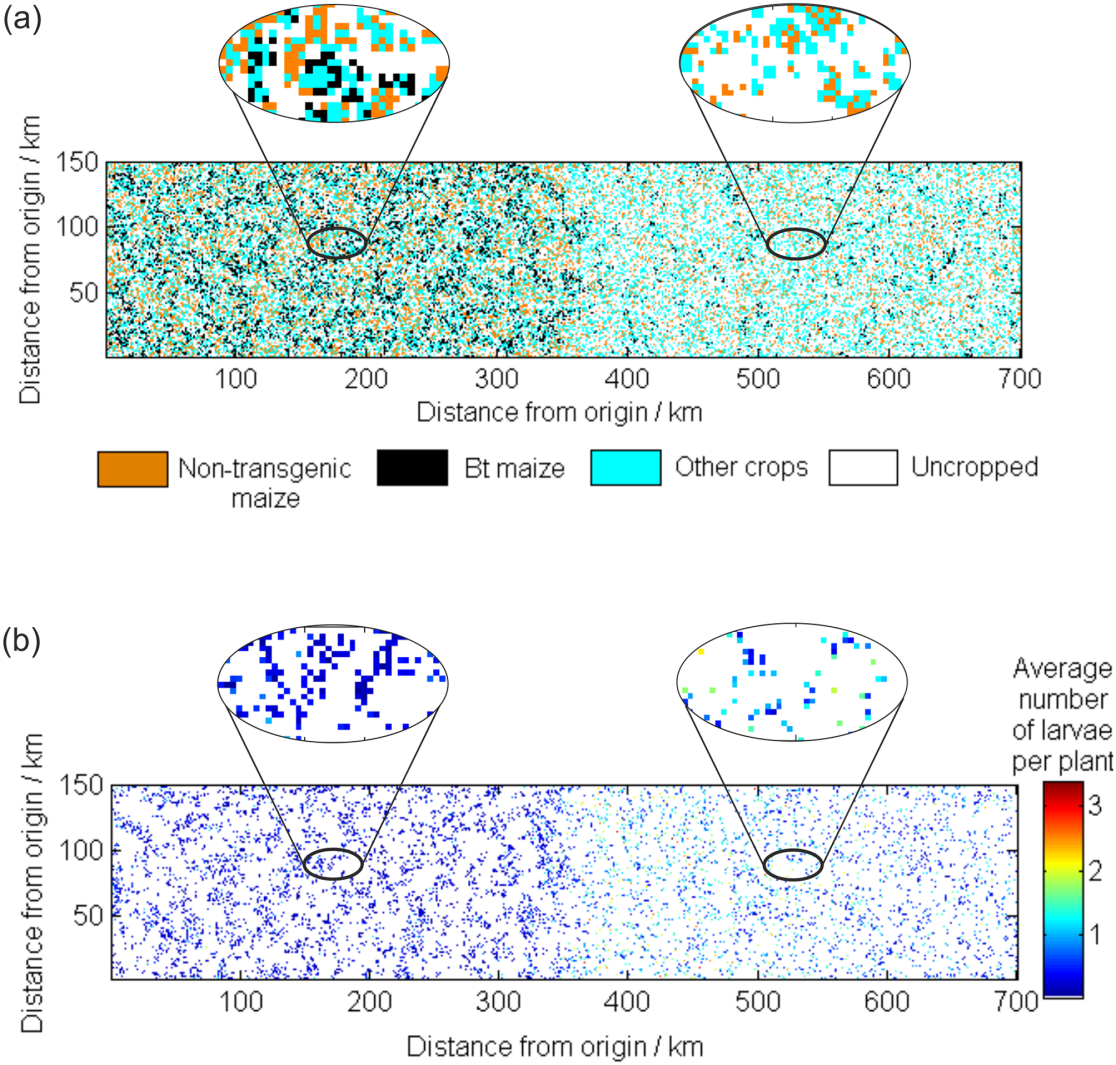
The spatial distribution of crops and larvae in a single year of the simulation. (a) The land use in year 73 of simulated landscape under the neighbor-network. The left half of the landscape represents Minnesota (abscissa from 0 to 350 km) and the right Wisconsin (abscissa from 350 to 700 km); (b) shows the corresponding average number of overwintering larvae per plant. Enlarged sections show the spatial distributions in more detail.

In the neighbor network the solution slowly converges to a state where the proportion of *Bt* maize is approximately 0.67 in Minnesota and 0.24 in Wisconsin (Fig 4c). The difference in adoption rate results because the neighborhood connections are stronger in Minnesota than in Wisconsin due to a greater density of farms in Minnesota. Indeed, in the simulated Wisconsin landscape, more farms are likely to be isolated and so have no neighbors growing *Bt* maize to compare profits with (see Fig 5a). Simulated ECB populations in Minnesota are lower than those in Wisconsin, where adoption of *Bt* maize was smaller (Fig 4d). Fig 5b shows the average number of overwintering larvae per plant in each cell for a single year of the simulation. The average numbers of larvae in Wisconsin reach larger levels, and even for isolated farms in Minnesota the pest is supressed by the larger amount of *Bt* maize grown in the surrounding area. For example between years 30 and 50 of the simulation shown in Fig 4 the maximum number of ECB in any cell was 8.12 larvae per plant for Wisconsin and 2.69 for Minnesota. The responsiveness of the farmer to loss (parameter *β*) affects the convergence rate with smaller values of *β* taking longer to converge.

Results from the simulation where farmers were more responsive to loss from conventional maize if they had experience of growing *Bt* maize (varying-response-network simulations) are shown in Fig 4e and 4f. The simulation illustrates that adoption of *Bt* maize is more rapid than that of conventional maize.

Table 1 lists the average losses ($ ha^−1^ year^−1^) across the landscape between year 20 and 100 under each simulation, and the average proportion of the maize that is *Bt*. Initial years were excluded to allow the simulation to stabilize. Losses (*L*) were calculated from a baseline whereby conventional maize was grown in an ECB-free landscape, i.e., *L* = *Y*_*L*_ *m*_*p*_+*F*, where *Y*_*L*_ is the yield loss caused by the ECB, *m*_*p*_ is the crop price and *F* is the technology fee. These results are based on 10 realisations of each simulation. The average proportions of *Bt* maize are similar across the networks ranging between 0.41 (when *β* = 0.0108) and 0.67 (when *β* = 0.0003). The standard deviation of the proportions of *Bt* maize were generally smaller for the less responsive farmers (*β* = 0.0003). For the values *β* considered, mean losses are least in the varying-response-network scenario and greatest in the neighbor-network scenario. We also simulated losses under scenarios where the proportion of *Bt* in the landscape was fixed at a given proportion, with the smallest simulated losses averaging 11 $ ha^−1^ year^−1^ with a proportion of *Bt* of 0.61. The sensitivity of our results to model assumptions is discussed in S3 Text.

**Table 1:** The average losses and the average proportion of the crop that is *Bt* between year 20 and 100 under each simulation according to communication network type and value of the parameter β, which changes the responsiveness of the farmer to loss. The standard deviations are given in parentheses.

Network type	Value of *β*	Loss/$ ha^−1^ year^−1^	Proportion of *Bt*
Landscape-network	0.0003	15.63 (0.182)	0.67 (0.073)
	0.0055	14.28 (0.302)	0.45 (0.319)
	0.0108	14.02 (0.216)	0.51 (0.312)
Neighbor-network	0.0003	30.02 (0.420)	0.50 (0.045)
	0.0055	27.51 (0.548)	0.51 (0.043)
	0.0108	27.64 (0.749)	0.50 (0.039)
Kaup-network	0.0003	17.15 (0.132)	0.58 (0.089)
	0.0055	16.12 (0.141)	0.43 (0.304)
	0.0108	15.96 (0.278)	0.42 (0.275)
Varying-response-network	–	13.90 (0.285)	0.56 (0.088)

### Comparison of the dynamics of farmer behaviour with data

**Fig 6:**
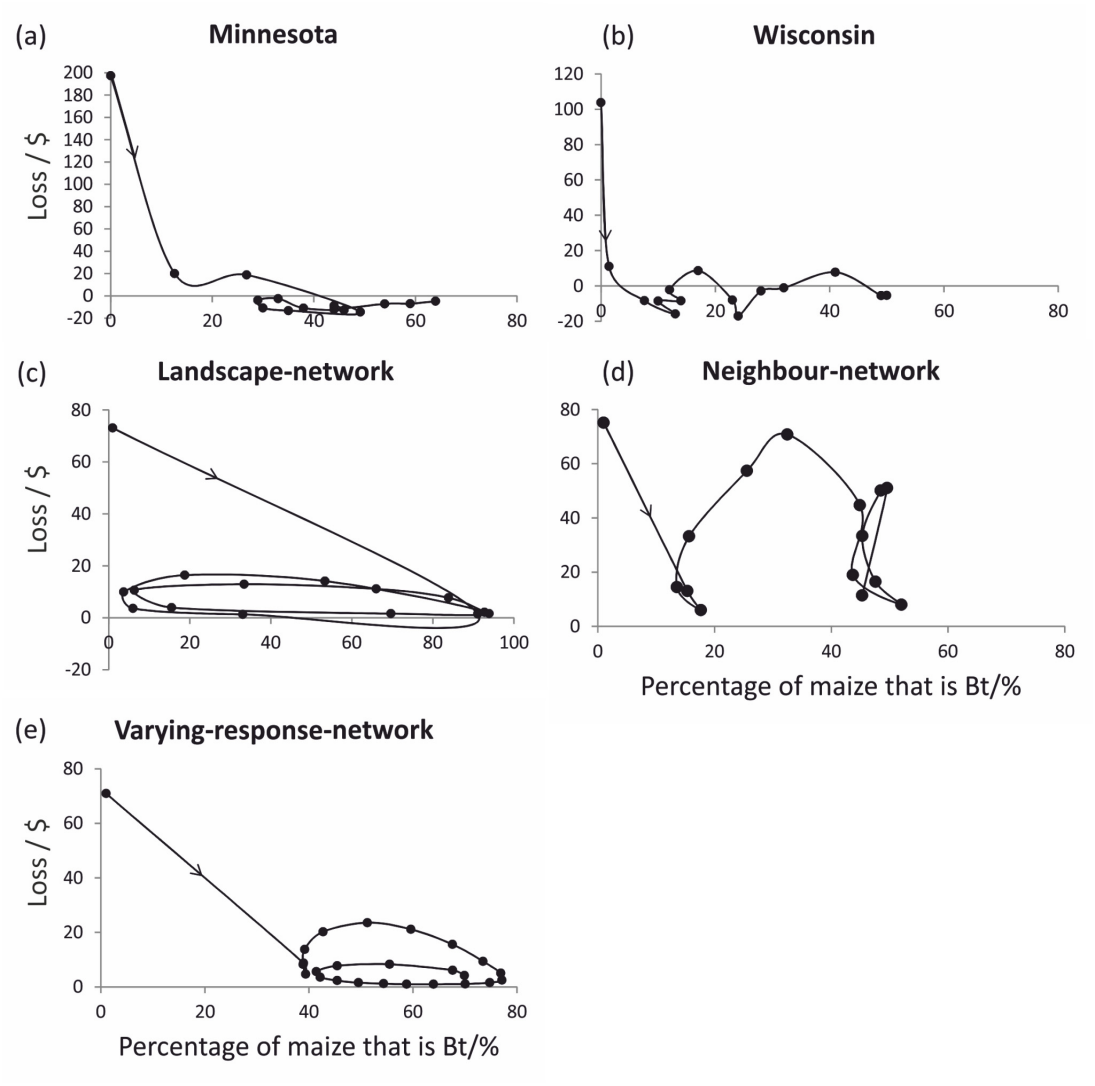
The loss in profit incurred by growing conventional maize compared with growing *Bt* maize plotted against the percentage of maize that is *Bt*. The arrow indicates the direction of time. Subplots (a) and (b) are based on data from states in the Corn Belt and subplots (c) to (e) are based on simulations.

To test the plausibility of the results from our model, we compared the observed and simulated dynamics of the relationships between loss incurred by growing conventional maize (calculated as above) and the percentage of maize that was *Bt* (Fig 6). The relationship between these two variables changes year on year depending on the corn borer population in the landscape. The dynamics observed in the data from Minnesota and the simulations for the varying-response-network are broadly similar (Fig 6a and 6e). The percentage of *Bt* maize grown increases until it is not profitable to grow *Bt*, then farmers start to move back to conventional maize only to return to *Bt* maize as losses increase later. The period of dis-adoption shown in Fig 6a is unlikely to be solely driven by the farmers’ perceptions of loss from corn borer infestation as it coincides with a period where there was a drop in confidence for the marketability of *Bt* maize, however our analysis gives support to the hypothesis that farmers’ perceptions of loss might explain dynamics. The Minnesotan data shows a second small drop in adoption over a two year period when the losses reach −13 $ ha^−1^ thereafter there is a steady increase in the percentage of *Bt* maize grown with no relationship to loss. Observed dynamics for Wisconsin show slower uptake of *Bt* maize compared with Minnesota (Fig 6b). This may reflect the fact that maize is grown on a much larger scale in Minnesota compared to other states including Wisconsin, which in turn may have implications for the way in which information is shared and how fields are managed in these states [33]. Similar to the neighbor network we also see that levels of *Bt* maize that initially control losses are subsequently less effective at the landscape scale and so the use of *Bt* is increased. No ECB resistance to *Bt* maize has been reported and so these changes in loss result from other factors such as climate or *N*. *pyrausta*.

## Discussion

Liu et al. [34] highlighted the importance of linking sociological influences to ecological systems. In our simulation we show how economic conditions can result in the suppression of a pest throughout a landscape. Our results accord with the findings of Bell et al. [2] who observed the impact of a coordinated response to ECB, and showed the planting of *Bt* maize in Minnesota led to a collapse in the cycle of ECB caused by *N*. *pyrausta*. In Wisconsin, however, where less *Bt* maize was grown, the cycle persisted. Similarly, Hutchison *et al*. [1] showed that farmers who grew conventional maize benefited from the area-wide suppression from *Bt* maize in the region. Our model shows a similar phenomenon, particularly exemplified in the neighbor-network simulation where a smaller proportion of *Bt* maize in Wisconsin resulted in a larger density of ECB compared with Minnesota, so that ECB population density continued to exhibit the *N*. *pyrausta* driven cycle. The landscape scale effects of the decisions made by individuals have been observed in other agricultural systems in which farmers’ decisions are influenced by social or economic factors or both and appear to be coordinated. The farmers’ behaviors results in substantial impacts on the population dynamics of species across landscapes. For example, Bianchi et al. [35] reported that coordinated changes in landscape composition negatively impact natural pest control, and Klein et al. [36] showed how agricultural intensification threatens wild bee pollination services at the landscape scale.

In our example, we show that decisions made by farmers on an individual basis impact ECB populations and the profitability of growing maize in the landscape. These decisions are driven by a range of external influences, from the advice of neighbors to information from extension specialists. We showed that the form of the network and the farmer responsiveness to loss substantially impact the dynamics of the system at all trophic levels. Generally we found that *Bt-*maize adoption oscillated in response to the prevalence of ECB in the landscape, and that the communication network and responsiveness of the farmer to loss influenced the amplitude and frequency of this oscillation. As the scale of communication networks increased so did the rate at which change occurred. This phenomenon was observed by Lambin et al. [37] who reported that rapid land-use changes often result when global influences replace local drivers. For example the global markets demand for certain commodities may rapidly change landscapes from longstanding diverse land-use patterns to more uniform cropping.

Of the networks we considered, the varying-response-network performed the best in terms of minimising losses and showed a reasonably constant proportion of *Bt* maize grown across time (Table 1). The farmers in this simulation had good access to information from across the landscape and were quicker to re-adopt *Bt* maize at the first sign of losses from ECB, yet slower to return to the more risky strategy of growing conventional maize. Importantly, our simulations show that to avoid extreme events some resistance to change must be inherent in the system. The varying-response-network did not outcompete the simulation with a fixed percentage of 61% *Bt* maize however. This outcome is compatible with the initial US-EPA resistance management requirements for ECB of at least 20% non-*Bt* maize planted each year, to serve as a refuge to maintain non-*Bt* selected susceptible moths in the landscape [1].

One aspect that we did not consider is that seed companies use market power to protect against the sales of *Bt* maize oscillating by selling the ECB-*Bt* maize seed bundled with other desirable seed traits and by reducing ECB-*Bt* maize prices so that farmers continue to buy the ECB-*Bt*-maize [38]. Similarly, seed dealers may promote *Bt* maize seed over conventional because they themselves receive a better rate of commission for *Bt* maize. The effect of such actions would be to inflate the reward farmers perceive is obtained from growing *Bt* maize, and so increase the adoption of *Bt* maize and drive the trajectories shown in Fig 6 to the right. Indeed any volatility in the price of seed or the harvested crop will impact the dynamics of the system. Increases in the price of maize or a reduction in the technology fee result would result in a lower tolerance to corn borer larvae. Another area not included in our analysis is the effect of farmer decisions on the evolution of resistance ECB to *Bt* maize. A recent review by Tabashnik et al.[39] found no evidence of a decrease in the susceptibility of ECB to Cry1Ab in *Bt* maize in the field. Others have used modelling to evaluate the effect of refuge planting strategies and including two or more toxins within a cultivar (pyramided toxins) on the rate of resistance evolution [22, 40–42]. These studies aim to guide regulatory policy designed to mitigate the threat of resistance. It is generally held that the greater the density of *Bt* maize in the landscape the faster the evolution of resistance. It follows that within the context of farmer behaviour, social factors that increase the use of *Bt* maize in the landscape would increase the rate of the evolution of resistance. Increased resistance of ECB to *Bt* maize would in turn result in farmers seeking alternative methods of control perhaps in the form of new toxins, or cropping strategies.

Our work has implications for other systems, whereby the ecology of a system is driven by individual decision makers following similar heuristics and experiencing similar influences. Examples include important systems where co-ordinated control can result in area-wide suppression of a pest or diseases. These systems typically involve insect pests that either cause damage to crops by herbivory (e.g. *Meligethes aeneus* F, *Spodoptera exempta Walker*) or act as a vector for disease [43]. The model framework presented here also has application to other areas such as disease prevention in a public health setting. There are clear parallels between landscape suppression of pests and diseases, and the herd immunity afforded when sufficient numbers of the population vaccinate. A number of modelling studies have been done to explore behaviour in the context of vaccination to try to understand the conditions that cause vaccine coverage to fall [44–46]. The conceptual difference between the vaccination studies and our study is that in our study the host of the insect pest is fixed in space and the insect moves across space, whereas in the case of human diseases the hosts move and transmit disease to one another. Our decision model was based on the farmers’ perceived profits. However, other social factors such as perceived food safety, the threat to non-target species and resistance management can effect decisions [47]. These factors often do manifest as economic factors but where they do not, they could be included in a model framework such as the one described by using opinion dynamics models [48]. Vaccination uptake is an example of a situation where often decisions are based on a perception of the safety rather than financial incentives (44). By understanding the dynamics of farmer decisions we can determine how to manage better the system, through improved communication, subsidy or taxation, to achieve robust and cost effective area-wide control, while minimizing the risk of the evolution of resistance to control strategies.

## Supporting Information

S1 Text The landscape model. (DOCX)

S2 Text The European corn borer model. (DOCX)

S3 Text Sensitivity of results from snow drift game to model assumptions. (DOCX)

S1 Fig The data used to support the decision model. The increase in area of *Bt* grown as a proportion of the area of non-transgenic maize between year *t* and *t+1* plotted against the net benefit of growing *Bt* calculated for year *t* (Hutchison et al., Science 2010; 330: 222).
(DOCX)

S2 Fig Results from Kaup-network simulation. (a) the percentage of maize grown in the landscape that is *Bt* and (b) the average number of larvae per plant across the two areas of the landscape. The parameter *β* = 0.0055.
(DOCX)

S1 Data Data on the maize yields, *Bt* uptake, average ECB per plant. Data on maize yields, *Bt* uptake and average ECB per year for Minnesota and Wisconsin with estimates of loss due to ECB. These data and calculations were reported in Hutchison et al., Science 2010; 330: 222.
(XLSX)
