# Supplementary material for: The Effect of Farmers’ Decisions on Pest Control with Bt Crops: A Billion Dollar Game of Strategy
Source: PLoS Comput Biol. 2015 Dec 31;11(12):e1004483. doi: 10.1371/journal.pcbi.1004483 (PMC4705107; doi:10.1371/journal.pcbi.1004483)
Supplement: S1 Text — (DOCX) [file pcbi.1004483.s001.docx]

**S1 The Landscape Model**

Our simulated landscape was parameterised so that it had similar attributes to the part of the Corn Belt that runs through Minnesota and Wisconsin. The landscape is modelled using a grid of 300 x 1400 cells. Each cell represents 25 ha (0.5km x 0.5km), similar to the typical size of maize fields in the region. We assume that one half of our modelled landscape is characteristically similar to Wisconsin and the other Minnesota. We simulated our landscape based on national statistics from 1997, 2002 and 2007 on county sizes, farms numbers, harvested areas and the area of maize grown [1, 2, 3]. We generated counties by sampling from distributions that characterised the observed county sizes. For Minnesota the county sizes were log-normally distributed with mean 160 560 ha and standard deviation 49 065 and for Wisconsin they were normally distributed with mean 180 426 ha and standard deviation 63 880. We randomly ordered the sizes of counties. We took the first county from the list and calculated how many grid squares it would occupy. Then assuming that the county had similar length and breadth, we allocated it to the top left hand corner of the landscape grid. Working through the list of counties we fitted each in adjacently making each as close to square as possible. Edge effects meant that this was not always possible and so in some cases the counties were more oblong.

Based on the available data, the number of farms per county was assumed to be 0.005 of the county size (ha). The national statistics categorised farms as harvesting either (i) 12.14–19.83 ha (ii) 20.23–40.06 ha (iii) 40.47–80.53 ha (iv) 80.93–201.94 ha (v) 202.34–404.28 ha (vi) 404.69+ ha. We calculated the proportion of farms in each category. The farms that harvested less than 12ha were excluded because each farm was less than half the size of our modelled field area (which is 25 ha) and they accounted for less than 2% of the total harvested area. We used an additive logistic distribution (see chapter 6 of Aitchison, [4]) to describe the farm size variable, and sampled from this distribution to generate the number of farms of each size in each county. In the model, the farm sizes were represented by 1, 2, 3, 5, 12 and 22 grid cells for Minnesota and 1, 2, 3, 5, 10 and 20 for Wisconsin. The larger farms in Minnesota were represented by more grid cells than those in Wisconsin so that the actual simulated harvested crop areas matched more closely the harvested crops in the survey data.

The sampled values for the number of farms of each size were used to calculate the total cropped area in each of the simulated counties. The difference between the sampled county area and the total cropped area is assumed to be un-cropped. In each county the cropped and un-cropped areas are fitted into the county randomly. Farms were fitted into the landscape so that they were approximately square using a similar method to the one we used to allocate the counties in the landscape.

Crops are assigned county by county. The proportion of corn grown in a given county was sampled from a beta distribution. The cropped cells were then allocated at random corn or other. For Wisconsin the mean and variance for the proportion of corn grown was 0.37 and 0.0066 respectively and for Minnesota the parameters mean and variance were 0.44 and 0.0062 respectively. Each year, the proportion of corn in a given county was resampled, and cropped cells were again allocated at random corn or other. This allowed for a proportion of fields to have rotations with maize crops grown consecutively and others to have a break crop that was not a host to the European corn borer.

**References**

1. United States Department of Agriculture (USDA).Census of agriculture. National Agricultural Statistics Service, Washington, DC. 2007. Available: http://www.agcensus.usda.gov/Publications/2007/Full_Report/
2. United States Department of Agriculture (USDA). Census of agriculture. National Agricultural Statistics Service, Washington, DC. 2002. Available: http://www.agcensus.usda.gov/Publications/2002/index.php.
3. United States Department of Agriculture (USDA). Census of agriculture. National Agricultural Statistics Service, Washington, DC. 1997. Available: http://www.agcensus.usda.gov/Publications/1997/index.php.
4. Aitchison J. The statistical analysis of Compositional Data. Chapman and Hall, London. 1986.
