# Supplementary material for: The Effect of Farmers’ Decisions on Pest Control with Bt Crops: A Billion Dollar Game of Strategy
Source: PLoS Comput Biol. 2015 Dec 31;11(12):e1004483. doi: 10.1371/journal.pcbi.1004483 (PMC4705107; doi:10.1371/journal.pcbi.1004483)
Supplement: S2 Text — (DOCX) [file pcbi.1004483.s002.docx]

**The European Corn Borer Model**

Our model follows the development and dispersal of ECB in a landscape. We assumed that the population was bivoltine and that generations did not overlap, as this is typical for the area of the Corn Belt in the US that we consider. The parts of the model that simulate the ECB development and transfer of infection of *Nosema pyrausta* are largely based on the models derived by Onstad et al. [1, 2, 3] and so we keep this part of our exposition brief. The description of the development of our dispersal model is novel and so more detailed.

**Egg fertility**

We assume that susceptible females produces approximately 235 fertile eggs and that infected females produce only a 30 percent of that number (based on [1, 2]). Infected males do not transmit disease to their off spring [4]. The infected females do however. Following Onstad and Maddox [2] we assume that the infection transfer rate from female to egg is 0.85.

**Larvae survival**

We made the simplifying assumption that the landscape comprises only (i) conventional maize crop (ii) BT maize and (iii) areas that are not habitable to the ECB. Similar to [1], we assume that in a non-transgenic maize crop 11.81% of the hatched larvae survive to instar 3 and this survival is density independent. Thereafter the density of the larvae is assumed to affect survival. The number of susceptible ($L_{5H})$or infected ($L_{5I})$larvae that survive through the third to fifth instars is modelled by

$$L_{5U}=\frac{\kappa L_{3U}}{1+{\lambda(L}_{3H}+L_{3I})} for U=H or I$$

(S2.1)

where $L_{3H}$ and $L_{3I}$ is the total number of susceptible and infected larvae respectively that survive to instar 3, and $\lambda$ and $\kappa$ are parameters with values of 0.9 and 0.656 respectively. The parameter $\lambda$ controls the carrying capacity of the host crop. For a single plant, data in [3] suggest a value of between 0.9 and 0.17 but at landscape scale we found this value too large because the density of corn in the landscape also affects the carry capacity of the host plants. Therefore we chose to use 0.067 as this value gave cycle amplitudes comparable to the observed data. The survival rate of infected larvae is assumed to be 51% of that of the susceptible larvae. The effect of the Bt toxin is modelled by scaling the number of larvae that reach instar 3 by 0.001 [5].

**Infection transfer**

Susceptible larvae may become infected if they come into contact with infected frass we estimated infection rate $R$ for each generation using

$R=1-exp(-\alpha L_{I3})$.

(S2.2)

Here $\alpha$ is a constant parameter and $L_{I3}$ is the number of infected third instar larvae.

**Overwintering**

We assumed that 8.1% of second generation larvae that are susceptible survive in the stalks through the winter [1], and that the proportion of infected larvae that successfully overwinter is assumed to be one third of that value [2].

**From pupae to adult moth**

The pupae survival is modelled as density independent. The mean survival rate is 0.89 [1]. Infected pupae have a mean survival rate of 0.63 [2]. Infected pupae do not pass on their infection to other moths. Fifty percent of emerging adults are assumed to be female [1].

The model described above can be expressed by the following set of difference equations:

$$\tilde{S}(t)=\frac{a\left( S\left( t \right)+cP\left( t \right) \right)e^{-\alpha P\left( t \right)}}{\nu+S\left( t \right)+P(t)}$$

$$\tilde{P}\left( t \right)=\frac{kP\left( t \right)+b(S\left( t \right)+cP\left( t \right))(1-e^{-\alpha P\left( t \right)})}{\nu+S\left( t \right)+P(t)}$$

$$S\left( t+1 \right)=\omega_{1}\frac{a\left( \tilde{S}\left( t \right)+c\tilde{P}\left( t \right) \right)e^{-\alpha\tilde{P}\left( t \right)}}{\nu+\tilde{S}\left( t \right)+\tilde{P}(t)}$$

$$P\left( t+1 \right)=\omega_{2}\frac{k\tilde{P}\left( t \right)+b(\tilde{S}\left( t \right)+c\tilde{P}\left( t \right))(1-e^{-\alpha\tilde{P}\left( t \right)})}{\nu+\tilde{S}\left( t \right)+\tilde{P}(t)},$$

(S2.3)

where *S*(*t*) and *P*(*t*) represent the number of susceptible and infected eggs for the first generation respectively and $\tilde{S}$(*t*) and $\tilde{P}$(*t*) are for the second generation. The first pair of equations describes the summer generation and the second pair the autumn-spring generation. The parameter values are α=0.9, *a*=1267.86, *b*=269.24, *c*=0.15, *k*=116.7161, ν=141.56, ω_1_=0.081 and ω_2_=0.02835.

**Dispersal**

We modelled the dispersal, mating and oviposition in four stages. First moths disperse, secondly they mate. The females then disperse again before oviposition. The dispersal functions represent the integration of the movement of moths over a period of days. In all cases the dispersal is modelled using a beta distribution to describe the proportion of moths at each distance. We chose the Beta distribution ($\mathrm{Beta}(\alpha, \beta))$because it gives a lot of flexibility in the position of the mode. The Beta distribution is defined on the interval $[0,1]$, but can be scaled as appropriate. In our model we assume dispersal is the same in all directions, and that at the boundary of the landscape the moths are reflected back. The proportion of moths landing (*p*_m_) in a particular grid cell is calculated by integrating the dispersal function over the area of the grid cell

$p_{m}=\alpha\int_{x=x_{L}}^{x_{U}} \int_{y=y_{L}}^{y_{U}} \left( x^{2}+y^{2} \right)^{\frac{\alpha}{2}-1}\{1-{\left( x^{2}+y^{2} \right)^{\frac{1}{2}}\}}^{\beta-1}dxdy$ (S2.4)

where $x_{U}$, $x_{L}$, $y_{L}$ and $y_{U}$ are the upper and lower bounds of the target grid cell scaled by dividing by the range of the scaled Beta function.

*Pre-mating dispersal* On the first night of enclosion females make an obligatory migratory flight [6, 7], and the distance covered is significantly larger than that covered by a male in a similar time. On the night that follows most females will be mated, and therefore pre-mating dispersal of females is likely to be achieved in one night. Males reach enclosure a few days before their female counter parts and so are likely to have moved reasonable distances before the females emerge [7]. In our model, we make the simplifying assumption that the probability density function (PDF) that describes the pre-mating dispersal of adult moths is the same for both sexes. Based on [6, 8, 9, 10], we assume that in spring the mode of the PDF is 10km and that 90% of the population travel less than 30 km. The dispersal of infected moths is reduced by 80%. In summer dispersal is more conservative. We assume a mode of 1km and that 90% of the adult moths fly less than 15km.

*Post-mating dispersal* Under typical conditions, the pre-oviposition period has a mean of 3.6 days [1]. Thereafter the mean oviposition period is approximately 10 days with oviposition decreasing with time. During this time a female could cover a considerable area. Based on [8] we assume that in spring the mode of the PDF is 35 km and that 90% of the population travel less than 60 km. The summer statistics are assumed to be mode 5 km with 90% of the population traveling less than 30 km.

**Analysis of the model without dispersal**

To investigate the sensitivity of the model behaviour to the parameter values we did a local sensitivity analysis whereby one parameter is changed at a time and the effect on the model behaviour observed. No information was available on the errors associated with the parameter estimates and so we explored ranges of values ±50 percent of the expected values. The results are shown in the Table S2.1.

**Table S2.1. The results of a local sensitivity analysis on the model of the European corn borer described by Equation (S2.3). Each parameter was adjusted by ±50% of its expected value and the effect on the dynamics of the solution observed. In all cases the solutions are oscillatory.**

| Parameter | New value | Average period | Average amplitude |
| --- | --- | --- | --- |
| *Standard values* |  | 6 | 6.0 |
| *a* | +50% | 5 | 7.3 |
| *a* | -50% | 17 | 2.3 |
| *b* | +50% | 6 | 7.9 |
| *b* | -50% | 6.66 | 5.5 |
| *c* | +50% | 6 | 6.1 |
| *c* | -50% | 7 | 6.1 |
| α | +50% | 6 | 5.0 |
| α | -50% | 6 | 6.6 |
| *k* | +50% | 6 | 5.6 |
| *k* | -50% | 7 | 6.8 |
| ν | +50% | 11 | 5.0 |
| ν | -50% | 5 | 5.9 |
| ω_1_ | +50% | 6 | 6.6 |
| ω_1_ | -50% | 9 | 6.0 |
| ω_2_ | +50% | 6 | 5.4 |
| ω_2_ | -50% | 6.5 | 6.5 |

**Analysis of the model with dispersal**

The model of the population dynamics in the landscape is affected by the proportion of maize in the landscape (i.e. the landscape carrying capacity). This is illustrated in Table S2.2 where the effect of different proportions of maize in the landscape on the model behaviour is reported. Note that in our model the proportions of crop area that are maize are sampled from distributions with expected values of 0.44 in Minnesota and 0.37 in Wisconsin.

**Table S2.2 The effect of different proportions of maize in the landscape on the dynamics of the solution of the corn-borer model with dispersal.**

| Proportion of maize | Average period of cycle | Average amplitude of cycle |
| --- | --- | --- |
| Saturated landscape where every cell grows maize (results the same as model without dispersal) | 6 | 6.0 |
| Landscape with all cropped area allocated to maize | 6 | 4.1 |
| Landscape with 75% cropped area allocated to maize | 6 | 3.0 |
| Landscape with 50% cropped area allocated to maize | 6 | 2.1 |
| Landscape with 25% cropped area allocated to maize | 6 | 1.2 |

**The effect of *Bt* maize on the population cycle**

Real data on the population of ECB shows that *Bt* maize damps the *N. pyrausta* cycle, and that the cycling can persist. The degree to which the cycle persists (in part) depends on the proportion of Bt in the landscape [11]. Below we show output from our model that results from a step increase of *Bt* maize in the landscape to 64% (Fig S2.1) and 45% (Fig S2.2). These illustrate that the model system exhibits under damping (where the *N. pyraustra* driven cycle in the ECB population persists) or over damping (where the cycle is extinguished). The stochasticity in the model means that although the first part of the simulation is run with no *Bt* maize the results are all slightly different.

Fig. S2.1: The proportion of Bt-maize is shown in red. It rises from zero to 0.64. The cycle in the ECB population (average number of over wintering larvae per plant — shown in blue) is no longer visible and so is *Overdamped*.

Fig. S2.2: The proportion of Bt-maize is shown in red. It rises from zero to 0.45. The cycle in the ECB population (average number of over wintering larvae per plant — shown in blue) is damped but persists. The cycle is *Underdamped***.**

**References**

1. Onstad DW. Simulation model of the population dynamics of *Ostrinia nubilalis* (Lepodoptera: Pyralidae) in maize. Environ Entomol 1988; 17: 969–976.
2. Onstad DW, Maddox JV. Modelling the effects of the microsporidium, *Nosema pyrausta*, on the population dynamics of the insect, *Ostrina nubilalis*. J Invertebr Pathol 1989; 53: 410–421.
3. Kang J, Onstad DW, Hellmich RL, Moser SE, Hutchinson WD, Prasifka JR. Modelling the impact of cross-pollination and low toxin expression in corn kernels on adaptation of European corn borer (Lepidoptera: Crambidae) to transgenic insecticidal corn. Environ Entomol 2012; 41: 200–211.
4. Zimmack HL, Brindley TA. The effect of the protozoan parasite Perezia pyraustae Paillot on the European corn borer. J Econ Entomol, 1957; 50: 637–640.
5. Pereira EJG, Storer NP, Siegfried BD. Inheritance of Cry1F resistance in laboratory-selected European corn borer and its survival on transgenic corn expressing the Cry1F toxin. Bull Entomol Res 2008; 98: 621–629.
6. Dorhout DL, Sappington TW, Rice ME. Evidence for obligate migratory flight behavior in young European corn borer (Lepidoptera: Crambidae) females. Environ Entomol 2008; 37: 1280–1290.
7. Hu Y. Dispersal and mating system of European corn borer, *Ostrinia nubilalis*, (Hübner) [Lepidoptera: Crambidae], in relation to Bt resistance management. PhD thesis, University of Minnesota. 2008.
8. Showers WB, Hellmich RL, Derrick-Robinson ME, Hendrix WH. Aggregation and dispersal behavior of marked and released European corn borer (Lepidoptera: Crambidae) adults. Environ Entomol 2001. 30: 700–710.
9. Worthley LH, Caffrey DJ. Spread and Infestation by the European corn borer during 1926. USDA Miscellaneous Circular NO. 104. 1927.
10. Dorhout DL, Sappington TW, Lewis LC, Rice ME. Flight behaviour of European corn borer infected with *Nosema pyrausta*. J Appl Entomol 2011. 135: 25–37.
11. Bell JR, Burkness EC, Milne AE, Onstad DW, Abrahamson M, Hamilton KL, et al. Putting the brakes on a cycle: bottom-up effects damp cycle amplitude. Ecol Lett. 2012; 15: 310–318.
