# Supplementary material for: The Effect of Farmers’ Decisions on Pest Control with Bt Crops: A Billion Dollar Game of Strategy
Source: PLoS Comput Biol. 2015 Dec 31;11(12):e1004483. doi: 10.1371/journal.pcbi.1004483 (PMC4705107; doi:10.1371/journal.pcbi.1004483)
Supplement: S3 Text — (DOCX) [file pcbi.1004483.s003.docx]

**S5 Sensitivity of Results from Snow Drift Game to Model Assumptions**

**The dispersal model**

Our model of the moth dynamics captures the observed dynamics of the systems but, in particular, the information available to parameterise dispersion was limited and somewhat conflicting. We have explored the effect of varying dispersal and shown that our general conclusions hold. One notable effect is the effect on area-wide suppression of the pest. Hutchison *et al*. [1] showed that farmers who grew non-transgenic maize were benefitting from the area-wide suppression effect of those cooperating to reduce the pest. Our model shows a similar phenomenon exemplified in the neighbor-network simulation where a smaller proportion of Bt in the Wisconsin part of the landscape resulted in a larger density of ECB compared with Minnesota. Increasing the dispersal kernel of adult moth makes the differences smaller and decreasing it makes them larger.

**The carry capacity of the modelled landscape**

Increasing the carrying capacity increases the amplitude of the oscillations in the population density of the moth and this in turn impacts the proportion of Bt that is needed to minimise loss. This has the effect of shifting the proportion of Bt that is grown up on the ordinate of Fig. 4.

**The landscape model**

Our modelled landscape assumes that farms are randomly distributed in the landscape. We did not include any spatial clustering of farms within the landscape. This will have negligible effect on the population dynamics of ECB because of the large distances it travels, but the configuration of farm neighbours in the landscape would have some impact on the neighbour network results: greater connectivity would reduce the likelihood of isolated farms and so, in particular, would increase the Bt uptake observed in our Wisconsin simulation.

**References**

1. Hutchison WD, Burkness EC, Mitchell PD, Moon RD, Leslie TW, Fleischer SJ, et al. Areawide suppression of European corn borer with Bt maize reaps savings to non-Bt maize growers. Science. 2010; 330: 222–225.
