# Supplementary material for: The Effect of Farmers’ Decisions on Pest Control with Bt Crops: A Billion Dollar Game of Strategy
Source: PLoS Comput Biol. 2015 Dec 31;11(12):e1004483. doi: 10.1371/journal.pcbi.1004483 (PMC4705107; doi:10.1371/journal.pcbi.1004483)
Supplement: S1 Fig — The increase in area of Bt grown as a proportion of the area of non-transgenic maize between year t and t+1 plotted against the net benefit of growing Bt calculated for year t (Hutchison et al., Science 2010; 330: 222). (DOCX) [file pcbi.1004483.s004.docx]

**Figure of the data used to support the decision model**

Goodness of fit

| Criterion | Exponential | Logistic |
| --- | --- | --- |
| R-Square | 0.384 | 0.329 |
| BIC | -28.2 | -25.6 |
| LogL | 29.7 | 28.7 |
