# Supplementary material for: The Effect of Farmers’ Decisions on Pest Control with Bt Crops: A Billion Dollar Game of Strategy
Source: PLoS Comput Biol. 2015 Dec 31;11(12):e1004483. doi: 10.1371/journal.pcbi.1004483 (PMC4705107; doi:10.1371/journal.pcbi.1004483)
Supplement: S2 Fig — (a) the percentage of maize grown in the landscape that is Bt and (b) the average number of larvae per plant across the two areas of the landscape. The parameter β = 0.0055. (DOCX) [file pcbi.1004483.s005.docx]

**S4 Figure of the results from Kaup-network simulation**
